# Improved shimming for fMRI specifically optimizing the local BOLD sensitivity

**DOI:** 10.1016/j.neuroimage.2009.08.010

**Published:** 2010-01-01

**Authors:** Evelyne Balteau, Chloe Hutton, Nikolaus Weiskopf

**Affiliations:** a Cyclotron Research Centre, Liège University, Belgium; b Wellcome Trust Centre for Neuroimaging, University College London, UK

**Keywords:** BOLD sensitivity, Shimming, fMRI, EPI

## Abstract

In functional MRI, magnetic field inhomogeneities due to air-tissue susceptibility differences may lead to severe signal dropouts and geometric distortions in echo-planar images. Therefore, the inhomogeneities in the field are routinely minimized by shimming prior to imaging. However in fMRI, the Blood Oxygen Level Dependent (BOLD) effect is the measure of interest, so the BOLD sensitivity (BS) should be optimized rather than the magnetic field homogeneity. The analytical expression for an estimate of the BOLD sensitivity has been recently developed, allowing for the computation of BOLD sensitivity maps from echo-planar images and field maps. This report describes a novel shimming procedure that optimizes the local BOLD sensitivity over a region of interest. The method is applied *in vivo* and compared to a standard global shimming procedure. A breath-holding experiment was carried out and demonstrated that the BS-based shimming significantly improved the detection of activation in a target region of interest, the medial orbitofrontal cortex.

## Introduction

In functional magnetic resonance imaging (fMRI), magnetic field inhomogeneities due to air-tissue susceptibility differences can lead to severe signal dropouts and geometric distortions in echo-planar images (EPI). Therefore the optimization of the field homogeneity, so-called shimming, is an important step preceding the imaging process, and various shimming techniques using linear and higher-order resistive shim coils have been developed (Blamire et al., 1996; de Graaf et al., 2003; Gruetter, 1993; Kim et al., 2002; Poole and Bowtell, 2008; Prammer et al., 1988; Webb and Macovski, 1991; Wilson et al., 2002). The common overall goal of current approaches is to calculate the corrective shim currents in order to compensate for field inhomogeneities over a region of interest (ROI) by minimizing the spatial standard deviation of the magnetic field. Global shimming methods use this approach to perform an optimization over the whole brain before data acquisition (Gruetter, 1993; Kim et al., 2002; Prammer et al., 1988; Webb and Macovski, 1991). Based on the same approach, the automated shimming technique by Wilson et al. (2002) goes towards a more localized strategy by preferentially optimizing the field homogeneity in specified areas according to the experimental hypothesis. With dynamic shimming, the concept of localized optimization is extended by calculating the optimal correction separately for each slice and updating the shim currents in real time for each slice (Blamire et al., 1996; de Graaf et al., 2003). A variant has been proposed recently that divides the optimization problem into a series of sub-volumes rather than into slices, and calculates corrective shim currents separately for each sub-volume (Poole and Bowtell, 2008).

In fMRI, the measure of interest is the Blood Oxygen Level Dependent (BOLD) effect. Therefore shimming should ensure a high BOLD contrast-to-noise ratio or BOLD sensitivity (BS). The BOLD sensitivity is only indirectly related to the spatial variation of the magnetic field, i.e. the field homogeneity (FH). Although a high field homogeneity provides a high signal intensity and a subsequently high BOLD sensitivity, the BS also depends on the local TE (including both the nominal echo time and echo time shifts due to local field gradients in the phase-encoding direction), which is non-linearly related to the field homogeneity (Deichmann et al., 2002, 2003; Weiskopf et al., 2006, 2007b). The analytical expression for an estimate of the BOLD sensitivity has been previously developed, allowing for the computation of BOLD sensitivity maps from EPI data and field maps (Deichmann et al., 2002, 2003). The effect of through-plane and in-plane field variations on the BS have been investigated and strategies for BS optimization have been proposed (De Panfilis and Schwarzbauer, 2005; Deichmann et al., 2002, 2003; Weiskopf et al., 2006, 2007b). The choice of optimal compensation gradients, spatial resolution and slice tilt allows for signal recovery from areas affected by signal loss. However, if the parameters are optimized for one specific region of interest (ROI) only, the optimization may even lead to a reduction in the BS for other areas (Weiskopf et al., 2006), potentially limiting its use for experiments targeting various brain areas.

In this paper, we describe a shimming technique that optimizes the BOLD sensitivity locally over an ROI, while ensuring sufficient overall field homogeneity in order to limit geometric distortion and signal loss across the whole brain. To our knowledge it is the first shimming technique that (a) directly optimizes the BOLD sensitivity and (b) performs the BS optimization locally by taking advantage of the full range of available first- and second-order shim gradients. The method is applied *in vivo* and compared to a standard global shimming procedure. The BS improvement is assessed by measuring the theoretical BS after shimming and by comparing the performance of both procedures in detecting signal changes resulting from a breath-holding challenge. Furthermore, in order to demonstrate the general applicability of the BS optimization procedure, we present the results of simulations which were performed for different sets of EPI acquisition parameters.

## Methods

### Theory of BOLD sensitivity

In fMRI experiments based on the BOLD effect, the theoretical local BOLD sensitivity (BS) is proportional to the effective local echo time and the local intensity in a T_2_⁎-weighted image (Deichmann et al., 2002). Although a high field homogeneity provides a high signal intensity and a subsequently high BOLD sensitivity, optimal FH does not necessarily provide the best BS due to the non-linear relation between field homogeneity, local TE and BS, as shown in Eqs. (1)–(3) below. In the following, a blipped echo-planar imaging (EPI) sequence is assumed with a positive phase encoding gradient for prephasing and negative gradient blips for stepping through *k*-space. As described in detail by Deichmann et al. (2002, 2003), De Panfilis and Schwarzbauer (2005) and Weiskopf et al. (2006, 2007b), field gradients related to susceptibility variations may alter both the effective echo time and the image intensity locally and therefore affect the local BS. The BS can be expressed as a product of three factors corresponding to the effect of field gradients in the three orthogonal imaging directions: BS = BS_0_ · *α*_PE_ · *α*_RO_ · *α*_SS_ where BS_0_ is the BOLD sensitivity in the absence of field gradients (BS_0_ = 100%), *α*_PE_ and *α*_RO_ are the factors corresponding to the in-plane field gradients *G*_PE_ and *G*_RO_ in the phase encoding and readout directions respectively, and *α*_SS_ is the through-plane factor corresponding to field gradients in the slice selection direction *G*_SS_. Using the formalism developed previously (Deichmann et al., 2002, 2003; Weiskopf et al., 2007b), these factors can be written as follows:

(1) $$\{\begin{array}{ll} \alpha_{\text{PE}}=\frac{1}{Q^{2}}\cdot \exp\left(-\frac{TE-TC}{T_{2}^{*}}\right) & \text{if}\;TC-\frac{TA}{2}\leq TE\leq TC+\frac{TA}{2}\text{,}\\ \alpha_{\text{PE}}=0 & \text{otherwise} \end{array}$$

with $Q=1-\frac{\gamma }{2\pi }\Delta t\cdot \text{FoV}\cdot G_{\text{PE}}$ and $\text{TE=}\frac{\text{TC}}{Q},$ where TC is the nominal echo time in the absence of field gradients (entered in the scanner's user interface), TE is the effective local echo time (including echo time shifts due to local gradients in the PE direction), TA is the duration of the acquisition window, *γ* is the gyromagnetic ratio, Δ*t* is the echo spacing and FoV is the field of view.

(2) $$\{\begin{array}{ll} \alpha_{\text{RO}}=0 & \text{if }\left|\gamma \cdot G_{\text{RO}}\cdot TE\right|>\frac{\pi }{\Delta x},\\ \alpha_{\text{RO}}=1 & \text{otherwise} \end{array}$$

where Δ*x* is the voxel size in the readout direction.

(3) $$\alpha_{\text{SS}}=\text{exp}\left(-\Psi^{2}\right)\;\text{with}\;\Psi =\gamma \cdot G_{\text{SS}}\cdot TE\cdot \frac{\Delta z}{4\sqrt{\ln(2)}}$$

where Δ*z* is the slice thickness (full width at half maximum or FWHM) for a Gaussian shaped RF excitation pulse. In principle, the slice profile, position and thickness may be affected by field inhomogeneities if *G*_SS_ adds to the slice selection gradient. However, due to the large RF pulse bandwidth commonly used for excitation (1022 Hz in this experiment), the amplitude of the slice selection gradient (∼ 12 mT/m) is much larger than the susceptibility gradient *G*_SS_ (≤ 60 μT/m) and the effect is negligible.

### Shimming procedures

Two shimming procedures were implemented and compared. The first procedure is a standard procedure optimizing the global field homogeneity (FH-based procedure), i.e. minimizing the spatial standard deviation of the magnetic field, as described by Kim et al. (2002). It makes use of calibrated field maps for each shim coil to estimate the optimal shim currents in terms of the field homogeneity. The second shimming procedure aims at optimizing the local BOLD sensitivity (BS-based procedure), i.e. the parameter of interest in fMRI. This procedure makes use of the same calibrated field maps as the standard procedure. The field gradients are estimated by numerical differentiation from the field maps acquired prior to the fMRI experiment. The BS is estimated using Eqs. (1)–(3) above and the shim currents are optimized to maximize the BS over the ROI. The optimization and all simulations unless otherwise specified use the imaging parameters of the EPI sequence employed in this study (see below) and a value of T_2_⁎ = 45 ms (Wansapura et al., 1999). To solve this non-linear optimization problem, an iterative conjugate gradient technique is applied. Since shim gradients opposing the phase-encoding (PE) gradient (here, in the *y* direction) lead to an increase of the local TE and therefore of the BS (Eq. (1)), the optimization of the BS is expected to favor a high *G*_PE_ component in the shim field. Therefore, the BS optimization requires further regularization in order to avoid excessive geometric distortions. To this end, a large background region was defined that included most of the brain, which should be on average well shimmed—in the following referred to as WSA. The regularization was ensured by optimizing the BS over the given ROI, while constraining the field homogeneity and especially the *G*_PE_ component in the WSA. The first step of the constrained BS-based procedure consists in optimizing the field homogeneity over the WSA, excluding the ROI, by using the standard FH-based procedure. Then the BS is optimized over the ROI, while ensuring that the magnetic field standard deviation over the WSA (including the ROI) is not greater than 180% of its initial value and the mean *G*_PE_ not greater than 2.5 Hz/pixel. The latter constraint limits the geometric distortions in the EPI image to approximately 5% compression (for the PE bandwidth = 47.35 Hz/pixel used in this study).

For both the FH-based and the BS-based procedures, hardware limits for the shim currents are taken into account. If the result exceeds the limits for one or more shim currents, the exceeding value is replaced by its maximum and kept constant, while the other shim currents are re-adjusted. However, this situation was never encountered when shimming with either procedure on our scanner.

The shimming procedures were implemented in C++ using the open-source libraries ITK (Insight Toolkit (Ibanez et al., 2003)), VTK (the Visualization Toolkit (Schroeder et al., 1998)) and FLTK (the Fast Light Toolkit, www.fltk.org) for image processing, visualization and creation of a user interface allowing for the selection of the ROI and the definition of the WSA for the BS-based shimming procedure. The ROI and WSA can be defined as rectangular or ellipsoidal volumes to fit the experimental requirements. The optimization runs as an automated procedure, limiting user input to the definition of the ROI and providing the optimal shim currents as outputs. These outputs are then copied by the user to the manufacturer's scanner interface.

### Breath-holding experiment to assess improvement in BOLD sensitivity

Although the BOLD sensitivity may be theoretically estimated from field maps, a breath-holding experiment was conducted (Kastrup et al., 2001) in order to empirically determine whether and to what extent the predicted improvement in BS is translated into a higher functional sensitivity in an actual fMRI experiment. For example, the predicted increase in BS could theoretically be masked by an increase in temporal noise that is not considered in the above BS model. Breath holding as a hypercapnic challenge reliably increases the cerebral blood flow and the BOLD signal, and is comparable to CO_2_ inhalation, as shown in previous studies (Kastrup et al., 2001). It has been recently used to assess BOLD sensitivity changes related to TE and spatial resolution in standard EPI (Weiskopf et al., 2007b) and *z*-shimming in spiral EPI (Truong and Song, 2008).

The breath-holding experiment comprised four sessions. Each session consisted of four blocks of breath holding after expiration (duration = 30 s) alternating with blocks of free breathing (duration = 45 s), beginning and ending with free breathing. The subject was cued at the beginning of each block by visual display. Relatively long breath holding/free breathing periods were chosen to maximize the BOLD signal changes (Kastrup et al., 1998) and to allow a steady breathing state to be reached. The sessions were acquired with either the FH-based technique (session A) or BS-based shimming technique (session B), following either an ABBA or BAAB acquisition order to avoid order and time effects. For each volunteer the ROI (average volume = 70 cm^3^) was defined to approximately cover the medial orbitofrontal cortex while the WSA (average volume = 1470 cm^3^) encompassed the whole brain (see ROI and WSA contours in Figs. 2 and 4). The BS-based shimming procedure optimized the BOLD sensitivity over the ROI with field homogeneity and *y*-gradient constraints over the WSA (including the ROI), while the FH-based shimming procedure optimized the field homogeneity over the WSA (including the ROI). The volunteers (*n* = 4, age 28–39 years, one female) gave written informed consent according to the guidelines of the local ethics committee.

### MRI data acquisition and analysis

The experiments were carried out on a 3 T head-only scanner (Magnetom Allegra, Siemens Medical Solutions, Erlangen, Germany) operated with the standard transmit-receive quadrature head coil. Shimming was performed with the three first-order gradients (*X*, *Y* and *Z*) and five second-order shims (*Z*^2^, *ZX*, *ZY*, *X*^2^–*Y*^2^ and *XY*). Functional MRI data were acquired with a blipped EPI sequence with the following parameters: TR = 2080 ms, TE = 30 ms, FoV = 192 × 192 mm^2^, 64×64 matrix, 32 transverse slices with 2 mm thickness and 1 mm inter-slice gap, flip angle = 90°, echo-spacing = 330 μs. These EPI acquisition parameters are standard parameters, routinely used in fMRI studies at 3 T (Bach et al., 2009; Corney et al., 2009; Krüger et al., 2001; Triantafyllou et al., 2005) and in previous studies related to BOLD sensitivity optimization (Weiskopf et al., 2006). Images were reconstructed using a generalized reconstruction method based on the measured EPI k-space trajectory for minimal ghosting (Josephs et al., 2000). A gradient-recalled sequence was applied to acquire two complex images with different echo times (TE = 4.92 and 7.38 ms respectively) and generate field maps. The difference between echo times was equal to one period of the water and lipid chemical shift difference at 3 T to reduce chemical shift effects. The other acquisition parameters were TR = 517 ms, FoV = 256 × 256 mm^2^, 64 × 64 matrix, 48 transverse slices with 4 mm thickness, flip angle = 90°, bandwidth = 260 Hz/pixel. Three field maps were acquired for each volunteer. In addition to an initial field map acquired for the calculation of the shim currents, two field maps acquired with either the BS-optimized or the FH-optimized shim currents were recorded at the end of the experiment for distortion correction of EPI data and calculation of the BS and effective TE maps after shimming. For each volunteer, the respiration was monitored with a breathing belt during the whole experiment and recorded using Spike2 and a CED 1401 interface (Cambridge Electronic Design, Cambridge, UK). In a separate session, a high resolution T_1_-weighted MDEFT image (Deichmann et al., 2004) was acquired for reference and creation of a gray matter mask for each volunteer.

All data analyses were performed using SPM5 (Wellcome Trust Centre for Neuroimaging, London, UK), the Brain Extraction Tool (BET, (Smith, 2002)), custom-made scripts in Matlab (The MathWorks, Natick, MA) and C++ implementation using the Insight Toolkit open-source library (Ibanez et al., 2003). EPI time series were corrected for motion and distortion using Realign and Unwarp (Andersson et al., 2001) together with the FieldMap toolbox (Hutton et al., 2002) in SPM5. For each volunteer all images (including field maps and T_1_-weighted MDEFT images) were coregistered, resliced and smoothed with a Gaussian kernel of FWHM = 5 mm. The time series of each voxel was high-pass filtered with a cut-off period of 150 s. A general linear model (GLM) was applied to the time series of each voxel (Worsley and Friston, 1995). Breath-holding blocks were modeled as a boxcar reference function derived from the respiration data and convolved with a Gaussian function (*σ* = 7.48 s) to account for the delayed and dispersed blood flow response (Weiskopf et al., 2007b). In order to reduce artifacts caused by head motion, covariates derived from head motion parameters were included in the GLM (Friston et al., 1996) as effects of no interest. Temporal autocorrelations were modeled by a first order autoregressive model. Voxels activated by the breath-holding challenge were determined by testing for significant positive correlation of the measured signal with the modeled response. *T* statistics were estimated for each voxel and activations passing a fixed voxel-wise threshold of *t *> 3.11 were considered significant (*p* < 0.001 uncorrected). Regression parameters of the GLM for the breath-holding effect were compared between BS-based shim sessions and FH-based shim sessions. Voxels showing a significant increase for the BS-based shim sessions compared to the FH-based shim sessions were determined using *T* statistics (*p* < 0.001 uncorrected).

BS maps and TE maps were estimated from the field maps. Changes in FH, BS and TE in the ROI and the WSA were analyzed. The improvement in BS was calculated as the relative difference in BS: 2⁎(BS_BS-based_ − BS_FH-based_)/(BS_BS-based_ + BS_FH-based_). Significance of the improvement in BS was determined by a Friedman's test (non-parametric repeated measures comparisons (Friedman, 1937)) comparing the data acquired using the BS-based and the FH-based shim. The FH, BS and TE changes predicted by the shim procedure were compared to the actual FH, BS and TE values achieved after shimming as determined from the field maps. For further exploration of the data, descriptive means and standard deviations (s.d.) were determined for each parameter across the ROI and WSA. In order to focus on gray matter voxels only and avoid partial volume effects, all maps (including statistical maps from the EPI time series analysis) were masked using a gray matter mask generated from the T_1_-weighted images.

### Simulations

In contrast to FH-based shimming, the BS-based procedure is influenced by the selected EPI acquisition parameters. Therefore, to explore a larger range of factors affecting the optimization and to demonstrate the general applicability of the technique, we performed simulations to assess the effect of varying the regularization constraints for different sets of EPI acquisition parameters and for ROIs defined in other regions of the brain (in particular in the left and right orbitofrontal cortices characterized by a unipolar *G*_RO_ gradient field, unlike the medial orbitofrontal cortex). The optimization was performed on real data acquired on subject #1, using Eqs. (1) to (3). The results of the simulations were analyzed in terms of predicted field homogeneity (including *G*_PE_, *G*_RO_, and *G*_SS_), BOLD sensitivity and effective TE. The standard EPI parameters were compared with alternative values for the nominal echo time (TC), echo spacing (Δ*t*), field of view (FoV) and slice thickness (Δ*z*). The behavior of the BS-based shimming was assessed by calculating the change in shim currents with respect to those obtained with the FH-based procedure.

## Results

### BOLD sensitivity estimated from field maps

**Fig. 1:**
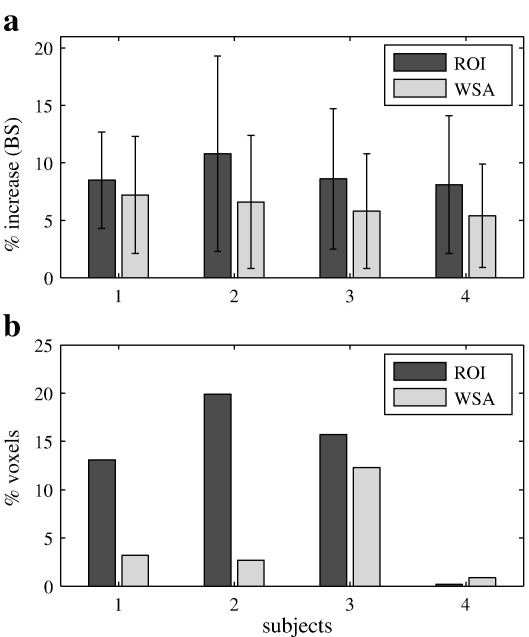
(a) BS percent increase (mean ± s.d.) observed with the BS-based shimming technique compared to the FH-based shimming technique. (b) Percentage of voxels showing a significant increase in sensitivity to the breath-holding activation with the BS-based shimming technique compared to the FH-based technique. All values estimated from gray matter voxels significantly activated by the breath-holding challenge only.

The BS-based shim significantly increased the BOLD sensitivity (BS) compared to the FH-based shim for all subjects (Friedman's test, *p* < 0.001). An increase between 8.1% and 10.8% was observed in the ROI and a global increase between 5.4% and 7.2% was observed in the WSA (including the ROI, Fig. 1a). The BS-based shim improved the field homogeneity in the ROI (field standard deviation reduced by 1.0% to 16.4% across the group) but decreased it in the WSA (including the ROI, field standard deviation increased by 83% to 103% across the group). An overall increase of the echo time of approximately 2 ms was observed with the BS-based technique compared to the FH-based technique. The FH, BS and TE maps theoretically predicted by the optimization procedure (before adjusting the shim currents) were in close agreement with the FH, BS and TE maps determined from the measured field maps after shimming (mean error less than 1%).

### BOLD sensitivity improvement assessed by a breath-holding experiment

**Fig. 2:**
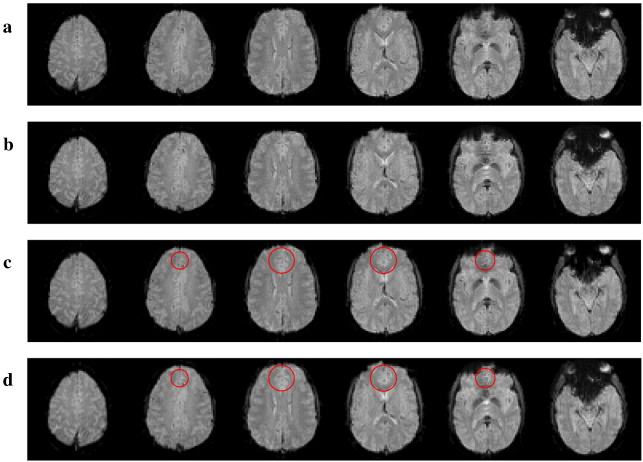
Transverse EPI slices from subject #1 acquired during the first FH-based shimming session (a, c) and the first BS-based session (b, d). Slices are displayed before (a, b) and after (c, d) undistortion. The ROI is overlaid in red on the undistorted images. Images are windowed identically.

**Fig. 3:**
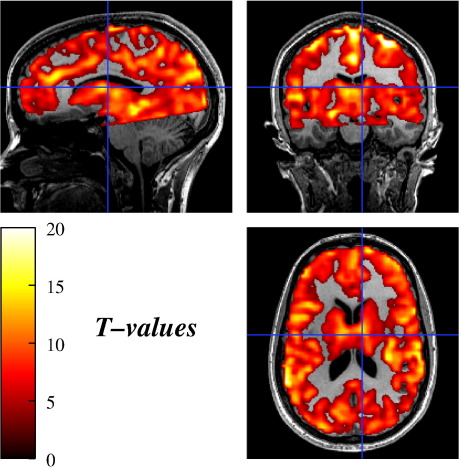
Statistical map of significant signal changes due to breath holding pooled across shimming methods (*t *> 3.11, *p* < 0.001 uncorrected, subject #4). The map is overlaid on the T_1_-weighted MDEFT image.

**Fig. 4:**
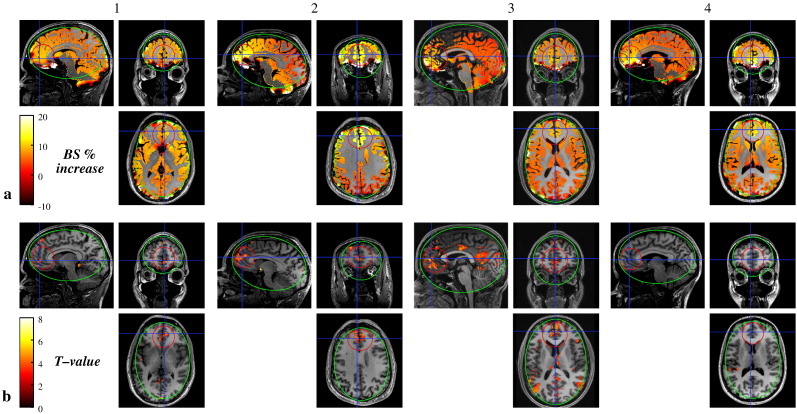
Local improvement of the estimated BS and the sensitivity to a breath-holding experiment with BS-based shimming. (a) Percent increase of the BOLD sensitivity in gray matter areas estimated from the field maps using the BS-based shimming compared to the FH-based shimming. (b) Statistical maps displaying gray matter areas that show significantly higher activation using the BS-based shimming compared to the FH-based shimming. The maps are displayed over the T_1_-weighted MDEFT image of each subject (#1–#4) and overlaid with the corresponding ROI (red) and WSA (green).

Fig. 2 shows the EPI images acquired with both shimming techniques before and after undistortion. Images acquired after BS-shimming are slightly compressed (Fig. 2b), with less than 5% compression. No significant differences in geometry were observed between both undistorted sets (Figs. 2c and d). The constraints on the field homogeneity and the *G*_PE_ shim component therefore guarantee the quality of the images. The breath-holding response overlaid on the T_1_-weighted MDEFT image is shown in Fig. 3 for subject #4. The overall breath-holding effect for both shimming methods was highly significant for all subjects (threshold *t* > 3.11, *p* < 0.001 uncorrected). The relative increase in BS and *T* statistics for the improvement in detecting a breath-holding response with BS-based shimming are displayed in Fig. 4. The relative increase in BS was more pronounced in the ROI as expected for a local optimization method (Figs. 1a and 4a). Only a few voxels at the inferior edge of the ROI showed a relative decrease in BS (less than 5% of the voxels in the ROI in three out of four subjects and 9.7% in subject #2). For subjects #1–#3 the increase in detected activation during BS-based shimmed sessions compared to FH-based shimmed sessions proved significant (*p* < 0.001, *t*-values > 3.11) in 13% to 20% of the gray matter voxels in the ROI (Figs. 1b and 4b). The number of voxels showing a significantly decreased activation did not exceed 0.5% in the ROI or 1.1% in the WSA for any subject. In subject #4, the BS-based shim did not lead to an improved detection of the breath-holding response.

Analysis of the respiratory data showed that the respiratory rate and breath-holding periods did not significantly vary between sessions and blocks for all subjects. The respiratory rates of subjects #1–#3 were close to their spontaneous breathing frequency (between 0.2 and 0.3 Hz) with breath-holding periods close to 30 s. However, subject #4 showed an unusually low breathing frequency (down to 0.09 Hz) when performing the task with significantly shorter breath-holding periods (20 s instead of 30 s).

### Simulations

**Fig. 5:**
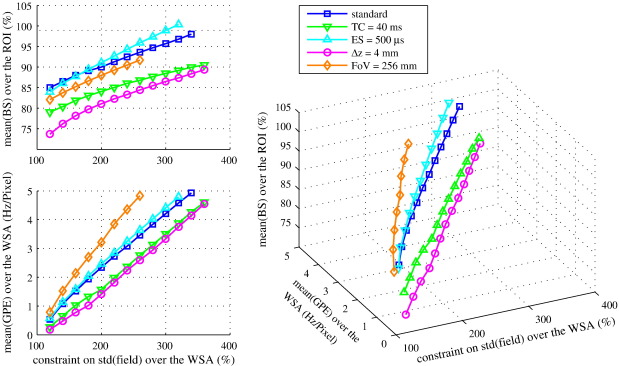
Effect of varying the EPI parameters and the optimization constraints. The mean BS achievable in the ROI and the mean *G*_PE_ observed in the WSA are reported as a function of the constraint on the field standard deviation. BS values are relative to BS_0_ = 100% in the absence of any field gradients. The standard EPI parameters were TC = 30 ms, Δ*t* = 330 μs, FoV = 192 mm and Δ*z* = 2 mm. Changing these parameters affected the result of the optimization, leading to relative change in the contributions of *X*, *Y* and *Z* shim components (Table 1) and in the maximally achievable BS. As an example, an increase of the FoV up to 256 mm (voxel size Δ*x* = 4 mm) will affect *α*_RO_ and *α*_PE_ with opposite effect. Simulations showed that the decrease in *α*_RO_ was almost exactly balanced by the increase in *α*_PE_. As a result, no BS improvement is observed compared to the standard EPI sequence.

Fig. 5 illustrates the effect of varying the regularization constraints for different sets of EPI parameters. In this figure the simulation results were based on the data from subject #1, using the same ROI and WSA as in the breath-holding experiment. No constraint on *G*_PE_ was applied. The graphs show how the estimated mean BS obtained in the ROI and the mean *G*_PE_ observed in the WSA change as the constraint on the field standard deviation is varied. The figure demonstrates that for this parameter space, there is a monotonic dependency of the optimal BS on both the field homogeneity constraint and on *G*_PE_, confirming that boundary conditions are required to avoid excessive image distortion and signal loss outside the ROI.

Table 1 shows the change in the shim currents simulated for the BS-based shimming relative to those obtained for the FH-based shimming for different sets of EPI parameters. The table shows simulation results based on the data from subject #1 using the same ROI and WSA as in the breath-holding experiment and allows the behavior of the BS-based shimming procedure to be characterized. For example, an increase of the TC from the standard value of 30 to 40 ms led to a larger change in the *Z* shim term and a smaller change in the *Y* shim term. This behavior can be explained by the increase of the influence of the factor *α*_SS_ on the optimization (from Eq. (3)) which leads to an increase in the *Z* shim term to reduce *G*_SS_ and a smaller change in the *Y* term which is related to *G*_PE_. Also as a result of increasing TC, there is more through-plane dephasing and hence the BS reduction is more pronounced. Equally, an increase of the slice thickness increased the impact of the *Z* shim. The change of the field of view or echo spacing did not have such a pronounced effect, but it affected which of the two boundary conditions (on field homogeneity or *G*_PE_) regularized the optimization.

The results of the simulations for different anatomical ROIs also demonstrated how the different field gradients within the ROI, *G*_PE_, *G*_RO_ and *G*_SS_, influenced the balance between the three BS factors *α*_PE_, *α*_RO_ and *α*_SS_ and hence the resulting BS (in particular in the WSA). As an example, the left orbitofrontal cortex exhibited a unipolar *G*_RO_ gradient which led to a larger contribution of *α*_RO_ in the BS optimization, and a larger contribution of the *G*_RO_ components in the resulting shim currents (data not shown). The BS gain estimated in the ROI was similar to the one observed with the medial orbitofrontal cortex but was smaller in the WSA due to the *G*_RO_ contribution.

## Discussion

This study introduces a novel approach to setting the shim currents for fMRI, directly optimizing the BOLD sensitivity (BS) instead of the field homogeneity (FH) as is conventionally done. The theory and implementation of the BS-based shimming technique is presented and validated using estimated BS maps and a breath-holding experiment. The use of first and second-order shim coils in the optimization process allows for a local optimization of the BOLD sensitivity. The ROI can be defined easily in an interactive user interface as rectangular or ellipsoidal volumes to fit the experimental requirements. The BS-based shimming technique significantly increases the BOLD sensitivity in the target ROI (the medial orbitofrontal in this study). Furthermore, it does not significantly decrease the sensitivity in any other brain area.

### Comparison with existing methods

Various techniques have been proposed previously to compensate for susceptibility artifacts in EPI and directly or indirectly optimize the BOLD sensitivity. By minimizing the field standard deviation and especially its through-plane component, the standard shimming techniques (Gruetter, 1993; Kim et al., 2002; Prammer et al., 1988; Webb and Macovski, 1991) reduce signal loss and indirectly increase the BS, even though the BS is not explicitly the target parameter. To our knowledge, this work incorporates for the first time the direct optimization of BS as the objective in shimming and thus outperforms the conventional shimming approaches for fMRI. Compared to existing methods for BS optimization that optimize the slice tilt (Deichmann et al., 2003), spatial resolution (Weiskopf et al., 2007b), phase-encoding direction (De Panfilis and Schwarzbauer, 2005; Weiskopf et al., 2006) or *z*-shimming gradient (Constable and Spencer, 1999; Deichmann et al., 2003; Glover, 1999; Merboldt et al., 2000; Weiskopf et al., 2006; Yang et al., 1997), the BS-based shimming method compensates susceptibility-induced gradients rather locally using higher-order shim coils. Since the conventional BS optimization methods use only linear compensation gradients and global strategies to compensate field gradients in the target ROI, they may exacerbate BS losses in non-target areas (Weiskopf et al., 2006). The constraints applied to the BS-based optimization presented here prevent this from happening.

Localized FH-based shimming techniques may also lead to severe signal loss and image distortions in areas outside the target ROI (Schneider and Glover, 1991). However, Wilson et al. (2002) have developed an automated shimming procedure that optimizes the FH preferentially over a target ROI while ensuring sufficient field homogeneity in the whole brain. Although this local FH-based optimization procedure led to an increase in BS in the target ROI, the optimization specifically targets the FH and improves the BS only indirectly. Since the BS depends non-linearly on the FH (Deichmann et al., 2002; Weiskopf et al., 2007b), the average BS in a region of interest may not be optimal even when the average field homogeneity is optimized as done by FH-based shimming. A comparison of the presented local BS-based optimization with a local FH-based optimization as described by Wilson et al. (2002) would be interesting but is out of the scope of the current study which focuses on the novel BS optimization itself.

The BS-based shim would benefit from dynamically updating the shim currents, in order to achieve a better global BS optimization from locally optimized shim currents (Blamire et al., 1996; de Graaf et al., 2003). With dynamic shimming the shim currents for each slice acquired are updated in real-time. Since the brain can be divided into subvolumes including voxels of similar field gradients which require similar compensations for an optimal BS (Weiskopf et al., 2006), the BS-based shimming would especially benefit from the recent concept of parcellated dynamic shimming (Poole and Bowtell, 2008) where the shim currents are optimized for each subvolume separately. Dynamic shimming requires hardware and software modifications that are not readily available for standard clinical MRI scanners. However, this limitation mainly applies to second-order shim terms. The linear *X*, *Y* and *Z* shim terms can generally be dynamically updated on standard MRI scanners. A dynamic update of these components, even for a simple slice-based parcellation of the volume, is expected to improve the BS gain based on a recent study using slice-dependent *z*-shim which was optimized using the BS model presented here (Weiskopf et al., 2007a).

In general the improvement in BOLD sensitivity due to the BS-based shimming technique can readily be combined with other BS optimization methods, such as *z*-shimming, optimized slice tilt, and optimized phase-encoding direction (Deichmann et al., 2003; Deichmann et al., 2002; Weiskopf et al., 2006, 2007b), to improve even further the BS in areas suffering from susceptibility artifacts.

### Considerations

The BS-based shimming procedure yields a local effect on the ROI but also a more wide-spread effect on the WSA. An important qualitative difference between the regions and between the shimming procedures results from the optimization of the *G*_PE_ to increase the local BS and its interaction with the optimization constraints. Compared to FH-based shimming, the BS optimization will usually lead to an increased *G*_PE_, as theoretically predicted from Eq. (1) (*α*_PE_) and observed in this study (Table 1). This increase in *G*_PE_ causes a relative increase of the local TE and a compression of the imaging voxel. Indeed, such an increase was observed in the orbitofrontal cortex ROI, bringing the local TE close to the nominal TE value (TC) and reducing the susceptibility-induced stretching of the voxels in this area while increasing the BS by ∼ 9% (Fig. 1a). For example in subject 1, compared to the FH-based shimming which yielded a 7.8% stretching in the ROI and TE = 27.1 ms, the BS-based shimming yielded only a 1.2% stretching in the ROI and TE = 29.4 ms, efficiently compensating for local susceptibility gradients (by use of first- and second-order shims, Table 1).

Further to this local ROI effect, we also observed a small but wide-spread voxel compression (∼ 4.1% in experimental data and simulations on subject #1, Figs. 2 and 5 respectively) and an increase of TE (from TE = 29.6 ms with the FH-based procedure to TE = 31.2 ms with the BS-based procedure) in the WSA. This change should theoretically increase the BS by ∼ 5% in areas without field inhomogeneities and may explain a significant amount of the BS increase observed in the WSA. Note that the same increase could also be achieved in perfectly shimmed areas by increasing the nominal TE and FoV in the PE direction. It is not surprising that a global change of these parameters can increase the BS in the WSA, since the nominal TC = 30 ms and voxel dimension of 3 mm is a trade-off between EPI parameters for perfectly shimmed areas and areas affected by susceptibility-induced gradients (Weiskopf et al., 2006), i.e. it is slightly sub-optimal for relatively well-shimmed areas such as the WSA. Although the BS increase is desired, the accompanying voxel compression needs to be limited in order to maintain a high spatial fidelity. Therefore, the optimization is regularized by ensuring that relative voxel dimension changes do not exceed 5%.

The regularization not only avoids excessive image distortion but also signal loss in areas not included in the ROI. Extensive simulations revealed that for the given susceptibility-induced field inhomogeneities and EPI parameters the BS is a monotonic function that increases when increasing the constraint related to the field standard deviation (Fig. 5). As previously reported (Balteau and Weiskopf, 2008), a tight constraint may overly limit the BS improvement, while no constraint leads to severe distortion and signal loss. Therefore, the constraints play an essential role and must be carefully chosen based on external criteria. The field standard deviation was limited to a relative increase of 180%, since this variation was commonly observed and accepted under global shimming conditions across a defined set of regions of interest (Wilson et al., 2002). The constraint on *G*_PE_ was chosen to allow distortion of no more than 5% in the phase encoding direction. Although we strongly recommend that undistortion is applied in the post-processing, we consider this level of overall compression/stretching to be just acceptable in standard group studies using typical spatial smoothing kernels. It should be emphasized that the BS-based optimization targets the mean of the estimated BS over the ROI. As a result, a few voxels in the ROI showed a relative decrease in BS. Alternative optimization schemes could be used that minimize any BS decrease even in single voxels using a different objective function (e.g. weighted mean).

The breath-holding experiment used to assess the increase of functional sensitivity reflects closely an fMRI experiment but still differs slightly. In particular, it is difficult to control for the breath-holding performance of the subjects, since extensive breath-holding periods are required. Here, one of the four subjects performed rather poorly, leading to short and variable breath-holding periods. This may explain why no significant increase in the breath-holding response was observed after BS-based shimming, although an improved BS was predicted by the shim procedure. Further, the challenging breath-holding task leads to head motion patterns that may be different from patterns typically observed in fMRI, affecting the temporal noise.

The ROI and WSA are defined by the user with the graphical user interface. This may lead to some variation of the anatomical definition and consequently shim performance across subjects in a multi-subject study. However, the ROI/WSA are usually relatively large, making the shim optimization rather insensitive to small mis-alignments/mis-definitions. Further, the current approach could be combined with an automatic registration and segmentation of the brain in pre-defined regions of interest (Wilson et al., 2002) to reduce the user-related variability in the shim. For example, a set of named problematic regions of interest could be defined independently on a template and subsequently selected by the user and included or excluded from the BS optimization.

There are a few situations where the BS-based procedure may not be preferable to a standard FH-based shimming procedure for fMRI studies. First, the BS-based method should not be used for fMRI studies requiring a high resolution and spatial accuracy if no image undistortion is applied, since the localization of the BOLD response may be incorrect due to increased *G*_PE_ values. However, in group studies that usually include spatial normalization and significant spatial smoothing, this may be of less concern. Second, to our knowledge, there is no established integrative model for estimating physiological noise in areas affected by susceptibility artifacts. As an approximation, our BS model does not take into account the effect of the physiological noise on the BS estimate and our results show that this approximation is effective in increasing the BS for the particular EPI parameters and field strength used here. However, as a precaution, we would recommend not to use this model without additional testing at higher SNR (resulting from e.g. larger voxel size or higher field strength) due to the relatively higher physiological noise component that would affect the accuracy of the BS estimate (refer also to following discussion on an integrative BS model). Third, the BS optimization is a non-linear problem, making it more sensitive to noise in the field maps and convergence to local minima. In particular, the size of the ROI should be large enough to achieve low noise estimates for the BS. With a small ROI and noisy field map data, discontinuities in the BS cost function can appear and the iterative conjugate gradient optimization method may fail. An alternative optimization algorithm like simulated annealing (Haddock and Mittenthal, 1992; Ikonomidou et al., 2005) may improve the convergence in these cases.

Although the optimization algorithm is fast (less than 2 min on the scanner console; Intel Xeon, 3.6 GHz, 4 cores, 2 GB RAM), several time-consuming steps could be modified to reduce the duration of the shimming procedure. Faster MRI sequences (e.g. EPI) could be used to acquire the initial field map, while 3D acquisition techniques would increase the signal-to-noise ratio. Currently, the optimized shim values must be copied manually to the manufacturer's user interface. Time would be saved and errors would be avoided if these values were directly applied to the system.

### Implementation on other systems

Although the technique has been implemented on a specific system and for a particular set of acquisition parameters, it can be readily adapted to other systems and other EPI protocols. Moreover the effect of changing the EPI acquisition parameters was investigated in this study using simulations based on the BS model. The results provide a qualitative description of the model behavior. In practice, the implementation of the technique on another system requires (1) the acquisition of a set of reference field maps characterizing the scanner's shim coils and (2) specifying the EPI sequence and system parameters, namely: nominal echo time (TC), spatial resolution, echo spacing, PE bandwidth and T_2_⁎ of the brain tissue. The reference field maps are acquired and pre-processed once and used by the optimization procedure to predict the effect of the shim currents. The reference set is system-dependent and a new reference set should be acquired if a major software or hardware upgrade is applied to the system. The other EPI and system-specific parameters are stored in a text file and can be easily adapted. The procedure can run on computers with relatively low specifications (a minimum of 1.66 GHz CPU and 1 GB RAM is recommended) and requires no additional software. Note that for a local optimization, second- or even higher-order shim coils should be available on the system.

### An integrative BOLD sensitivity model: perspectives

At 3 T, the physiological noise can dominate the other noise components and affect the gain in SNR expected at higher field strength (Krüger and Glover, 2001; Krüger et al., 2001; Triantafyllou et al., 2005; Van de Moortele et al., 2008; Wu and Li, 2005). The BS model used in this experiment assumes a simplified, constant noise model independent from the optimization parameters. However, recent noise models show that the relative physiological noise contribution depends on the thermal SNR and thus on field strength, signal intensity and TE (Krüger and Glover, 2001; Krüger et al., 2001; Triantafyllou et al., 2005; Van de Moortele et al., 2008; Wu and Li, 2005). Since the physiological noise increases when increasing the local TE and the signal intensity, it will be affected by shimming. So far these models are not directly applicable to BS shim optimization, since they do not take susceptibility and off-resonance effects into account. In the future, the accuracy of the BS estimate may benefit from an integrative approach combining models of all noise sources and susceptibility-related BS changes.

With the EPI parameters used in this experiment (3 × 3 × 2 mm^3^ resolution, 90° flip angle) thermal noise dominates over the physiological noise with a physiological-to-thermal noise ratio of approximately 0.89 (Triantafyllou et al., 2005). In this regime dominated by thermal noise, our results suggest that the simplified noise model used for BS optimization is still a good approximation and would also work for higher spatial resolution and lower flip angle (i.e., lower SNR). However, neglecting the physiological noise still affects the accuracy of the BS estimate. Qualitatively, an increase of the local TE and/or signal intensity leads to a relative increase of the physiological noise and limits the maximally achievable BS. In particular, the physiological noise is expected to penalize an excessive compression in the image (increase in voxel size) due to the increase in the *G*_PE_ field gradient component (decrease of the factor *Q*, increase of the factor *α*_PE_) and lead to a stronger contribution from the factors *α*_RO_ and *α*_SS_. The resulting BS gain is expected to be smaller than estimated in this study. This refinement of the BS model may be particularly relevant for high SNR EPI acquisitions (large voxels, ultra high fields, etc.) and further developments of the technique are under investigation. In practice, the implementation and use of an integrative BS model require the estimation of the noise components' standard deviation and correlation factors (Wu and Li, 2005). These parameters are system- and sequence-dependent, but seem to be relatively consistent across a group of healthy volunteers, and can be estimated from a multi-echo fMRI experiment as described by (Wu and Li, 2005). Either single-subject estimates from a pre-scan experiment or group mean values could be used, the latter reducing the duration of the procedure.

## Conclusion

We have presented and validated a method for improved shimming in fMRI. It directly optimizes the BOLD sensitivity, unlike previous approaches targeting the field homogeneity. The method is fast and operator-independent and can be readily combined with other methods for BOLD sensitivity recovery in areas affected by susceptibility-induced field inhomogeneities.

## Figures and Tables

**Table 1:** Relative variation in the shim currents obtained with the BS-based procedure compared to the FH-based procedure.

	*X*	*Y*	*Z*	*Z*^2^	*ZX*	*ZY*	*X*^2^ –*Y*^2^	*XY*	BS⁎	std(F)	*G*_RO,*X*_	*G*_PE,*Y*_	*G*_SS,*Z*_	TE
	(μT/m)			(μT/m^2^)					(%)	(Hz)	(Hz/mm)			(ms)
*FH-based shimming*														
Standard EPI	–	–	–	–	–	–	–	–	77.3	36.1	0.13	-1.23	-2.05	27.7
Δ*t* = 500 μs									78.9					26.9
Δ*z* = 4 mm									68.8					27.6
FoV = 256 mm									77.3					27.1
TC = 40 ms									75.5					37.0

*BS-based shimming*														
Standard EPI	-0.0	22.5	-7.9	-1.0	1.7	24.1	-32.3	0.7	88.6	31.2	0.13	-0.19	-1.78	29.4
Δ*t* = 500 μs	-0.3	23.3	-4.8	-1.2	1.7	24.3	-32.4	0.8	89.0	32.3	0.11	-0.15	-1.91	30.2
Δ*z* = 4 mm	0.5	15.1	-22.4	0.1	1.7	23.1	-32.0	0.4	79.2	26.6	0.15	-0.50	-1.17	28.9
FoV = 256 mm	0.5	23.1	-5.6	-1.6	1.7	23.7	-32.3	1.7	86.2	32	0.15	-0.16	-1.88	29.7
TC = 40 ms	-0.5	18.6	-17.1	-0.6	1.7	23.3	-31.8	0.3	82.5	28.1	0.11	-0.36	-1.39	39.0

The effect of changing the EPI parameters is illustrated. The predicted field standard deviation, BOLD sensitivity, field gradients and effective TE are reported in each case (mean over the ROI). The BS and the effective TE estimates depend on the EPI parameters. The standard EPI parameters were TC = 30 ms, Δ*t* = 330 μs, FoV = 192 mm and slice thickness Δ*z* = 2 mm.

⁎ BS values are relative to BS_0_ = 100% in the absence of any field gradients.
